# Gender disparities in the mediating role of symptom knowledge level in reducing acute coronary syndrome (ACS) decision delay: Findings from a community-based study in China

**DOI:** 10.1186/s12873-023-00916-5

**Published:** 2023-12-16

**Authors:** Siyue Wang, Junxian Song, Chongyou Lee, Jin Jiang, Mengying Wang, Dongjing Liu, Zhuqing Wang, Yuan Yuan, Wenyong Li, Ren Zhou, Hongchen Zheng, Jianmin Wei, Yonghua Hu, Tao Wu, Zhenbiao Tian, Hong Chen

**Affiliations:** 1 Peking University Health Science Center, Beijing, China; 2 Center for Cardiovascular Translational Research, Peking University People’s Hospital Beijing, Beijing, China; 3 Beijing Red Cross Emergency Rescue Center, Beijing, China; 4 Institute of Reproductive and Child Health/Key Laboratory of Reproductive Health, National Health Commission of the People’s Republic of China, Beijing, China

**Keywords:** Acute coronary syndrome, Pre-hospital delay, Decision delay, Knowledge of prodromal symptoms

## Abstract

**Background:**

Implementing training programs to educate patients on the prodromal symptoms of acute coronary syndrome (ACS) may assist patients in accurately recognizing these symptoms, and ultimately decrease their time delay in seeking emergency medical services (EMS). However, the effectiveness of this approach remains uncertain, particularly among the Chinese population.

**Methods:**

A cross-sectional study was conducted within 22 communities in Beijing, China between 2015 and 2018, with a total of 1099 participants recruited. The study utilized a standardized questionnaire to evaluate the presence of intentional decision delay in turning to EMS under a hypothetical chest pain, the participants’ knowledge of ACS prodromal symptoms, and whether they had ever received any training programs aimed at increasing their symptom knowledge. Mediation analysis was performed with regression models and bootstrapping methods, and gender difference was further analyzed through moderated mediation analysis.

**Results:**

A total of 1099 participants (58.2% female, median [IQR] age 34 [20]) were included in the study. The results of the mediation analysis indicated that training programs were associated with a decrease risk in decision delay, with increased knowledge playing a mediating role (mediation effect/total effect = 36.59%, *P* < 0.0001). Gender modified this mediation effect, with it being observed only in the male group. Specifically, training programs were not found to significantly decrease decision delay among females (*P* > 0.05), even though they did improve women’s knowledge of ACS prodromal symptoms (β = 0.57, *P* = 0.012).

**Conclusion:**

The results suggested a relationship between prior training programs and reduced decision delay, with increased knowledge of prodromal symptoms of ACS serving as a mediator. However, the effect was only observed in male participants and not in female participants. This highlights the notion that mere transfer of knowledge regarding ACS prodromal symptoms may not be sufficient to mitigate decision delay in the female population. Further research is needed to corroborate these results and to gain deeper insights into the gender-specific barriers encountered in this study.

**Supplementary Information:**

The online version contains supplementary material available at 10.1186/s12873-023-00916-5.

## Introduction

The prompt and effective treatment of acute coronary syndrome (ACS) is crucial to patient outcomes [1–3], as research has shown that each 30-minute delay in treatment increases the mortality risk by 7.5% [4]. The COVID-19 pandemic has further exacerbated this problem by hindering proper emergency medical services (EMS) [3]. Despite progress in reducing in-hospital delay, little improvement has been seen in total treatment delay [5, 6]. This highlights the importance of addressing decision delay, which occurs when patients delay seeking professional health care since the onset of symptoms, and is a well-documented modifiable factor that can contribute significantly to total treatment delay [7–9]. Therefore, exploring ways to reduce decision delay is a crucial aspect of improving patient outcomes in ACS.

It’s hypothesized that knowledge of attributing cardiac prodromal symptoms of ACS is the key prerequisite to initiate instant responses in case of ACS events [10, 11]. To this end, various training interventions were carried out in communities with the goal of reducing decision delay by enhancing knowledge of ACS prodromal symptoms [12]. In this context, knowledge was regarded as a mediating factor linking training programs to decision delay [13]. Nevertheless, the evidence regarding the efficacy of these interventions is inconclusive, with some studies yielding conflicting results [12, 14–16]. Therefore, further investigation is needed to establish whether knowledge of prodromal symptoms indeed mediates the impact of training on decision delay, which could help inform the development of culturally appropriate and effective strategies to reduce decision delay in patients with ACS.

Gender differences in decision delay are well documented [17–19]. These differences have also been associated with disparities in short- and long-term outcomes of ACS between men and women [18, 20]. However, the role of gender in the mediation process remains unclear. Recent studies have suggested that although women can be trained to attain higher levels of ACS symptom knowledge, they are less likely to take immediate action in an ACS emergency [21]. Hence, it is crucial to understand these gender differences in order to design targeted public health interventions that address gender-specific barriers.

The purpose of this community-based retrospective cohort study was to examine the mediation of knowledge of ACS prodromal symptoms in the relationship between ACS training and decision delay, and to explore the potential modification of this relationship by gender, in a Chinese population. The study aimed to provide insights for future public health interventions by exploring the complex relationship between training, knowledge, decision delay, and gender.

## Methods

### Study design

The study aimed to assess the mediation effect of knowledge of attributing cardiac prodromal symptoms of ACS on the relationship between training and decision delay, and to explore the potential modifying effect of gender. The protocols adhered to the principles of the Declaration of Helsinki and were reviewed and approved by the Institutional Review Board of Peking University People’s Hospital. The study was conducted in 22 communities in Beijing, China over the period of 2015–2018.

### Study setting and population

The study population consisted of residents of the selected 22 communities in Beijing, China who were eligible for participation and consented to take part in the questionnaire survey. The selection criteria for the investigation units included stable population size, adequate support for the program, access to basic medical facilities, and an absence of overlapping interventions or media promotions. The study sites were strategically selected to represent a diverse range of urban and rural areas in the capital city, Beijing.

### Study protocol

A structured questionnaire survey was administered to gather information regarding the participants’ past training experience on ACS events, ACS prodromal symptom knowledge, response measures in the event of a hypothetical chest pain scenario, and additional demographic information. The correlation between past participation in ACS training programs and decision delay was evaluated. Subsequently, a mediation analysis was conducted to determine if the knowledge of prodromal symptoms mediates the relationship between training and decision delay. Additionally, the impact of gender on the mediation was assessed through a gender-stratified mediation analysis.

### Measurements

#### Exposure to past ACS training program

The participants’ exposure to past ACS training programs was assessed using a questionnaire. The questionnaire aimed to gather information on any education received on recognizing cardiac symptoms or other campaigns aimed at reducing decision delay. The specific questions used in the questionnaire can be found in the supplementary file (Supplementary File 1).

#### ACS prodromal symptom knowledge

The ACS Response Index Questionnaire was used to measure the participants’ symptom knowledge was adopted from a previously validated Chinese version [22]. We further incorporated the investigation into their hypothetical response behaviors in response to each symptom. The questionnaire was reviewed by professional emergency department cardiologists and epidemiologists to ensure its validity and reliability. The participants were asked to respond to questions about their knowledge of ACS prodromal symptoms, response measures under a hypothetical chest pain, and anticipated barriers for reluctance in seeking instant medical services. The detailed items for each of these domains can be found in the Supplementary file. Prodromal symptoms were defined as 7 items indicative warning signs that suggested ACS, both typical and atypical. The surveyed participants were expected to correctly identify all these 7 items suggestive of ACS. The total knowledge score was calculated by summing the number of correctly identified items. A higher score indicated a better understanding of ACS prodromal symptoms. The knowledge score was used as the mediator variable in the mediation analysis.

#### Decision delay

In the current study, decision delay was defined as the failure to seek immediate medical attention, such as calling for EMS or going directly to the hospital, in the event of a hypothetical chest pain. It was hypothesized that participants who indicated a lack of willingness to seek immediate medical services in the questionnaire would be at higher risk for decision delay. The questionnaire assessed the participants’ willingness to seek immediate medical services in the scenario where they experienced an abrupt chest pain lasting for over 5 minutes or where nitroglycerin-like drugs failed to relieve the pain.

### Statistical analysis

To ensure data quality, participants who were missing on gender information or decision delay options or had over 10% missing data were excluded from the analysis. Descriptive analysis was performed on categorical variables, which were presented as counts and percentages, and on continuous variables, which were presented with their corresponding statistical descriptions, either mean ± SD or median ± IQR, taking into account their distribution. Mediation analysis was conducted to assess whether knowledge of ACS prodromal symptoms played a mediating role in the relationship between exposure to past ACS training programs and decision delay, controlling for gender, age, education, occupation, and monthly income. Stratified analyses by gender were performed to examine potential modification effects on the mediation. The results of the statistical analyses were reported as point estimates with 95% confidence intervals (CI). The bootstrapping method with 1000 resamples was applied to test the significance of the mediation and moderated mediation analyses. The level of statistical significance was set at 0.05 and all tests were two-tailed. The data was analyzed using R software (Version 3.5.2).

## Results

### Demographic characteristics of the surveyed

A total of 1099 individuals from 22 communities participated in the survey, with 928 individuals included in the final analysis after excluding participants with missing gender or decision delay information (*n* = 11, 1.0%), or over 10% missing data (*n* = 160, 14.7%). The demographic characteristics of the participants (Table 1) showed that 58.2% of the participants were female and the median age was 34 with the majority having attained undergraduate education.

**Table 1:** The demographic characteristics of the participants surveyed

		N, percentage	*P*
Gender			
	Male	388 (41.8)	< 0.001
	Female	540 (58.2)	
Education			
	≤High	232 (25.0)	< 0.001
	Undergraduate	562 (60.6)	
	≥Postgraduate	134 (14.4)	
Age			
	18–25	172 (18.5)	< 0.001
	26–35	262 (28.2)	
	36–45	263 (28.3)	
	≥45	231 (24.9)	
Occupation			
	Retired	87 (9.4)	< 0.001
	Self-employed/Unemployed	27 (2.9)	
	Manager	168 (18.1)	
	Worker	190 (20.5)	
	Service	78 (8.4)	
	Technical	166 (17.9)	
	Else	212 (22.8)	
Income (Yuan/Month)			
	≤3000	205 (22.1)	< 0.001
	3000-5000	322 (34.7)	
	5000-10,000	267 (28.8)	
	≥10,000	134 (14.4)	
Medical Insured			
	Yes	897 (96.7)	< 0.001
	No	31 (3.3)	
Prior exposure to training programs			
	Yes	145 (15.6)	< 0.001
	No	783 (84.4)	
Ever witness ACS in families			
	Yes	243 (26.2)	< 0.001
	No	685 (73.8)	
Ever personally experienced ACS			
	Yes	97 (10.5)	< 0.001
	No	831 (89.5)	
Decision Delay			
	Yes	749 (80.7)	< 0.001
	No	179 (19.3)	

Approximately half of the participants (42.5%) had been exposed to training programs that aimed at increasing their ACS prodromal symptom knowledge. The results showed that 80.7% of the participants demonstrated intentional decision delay, among which a majority (67.5%) indicated a preference to wait until their symptoms subsided. In contrast, only 19.3% of participants stated that they would seek instant medical services.

### Knowledge of ACS symptoms

The average knowledge score of participants regarding ACS prodromal symptoms was found to be 3.81 out of 7, which corresponds to an accuracy rate of 54.4%. Results indicated a significant association between gender and ACS knowledge, with females exhibiting higher knowledge levels, both in terms of typical and atypical symptoms (*P* < 0.001). Additionally, it was found that younger age, higher income and education were associated with higher knowledge level of ACS symptoms (*P* < 0.05). A detailed distribution of ACS symptom knowledge can be found in the Supplementary File 1.

### Mediation analysis

#### Model 1: Total effect between prior participation in ACS training programs and decision delay

The results of Model 1 indicated a significant relationship between prior participation in ACS training programs and reduced intentional decision delay. The odds of intentional decision delay were 0.65 times lower (OR = 0.65, 95%CI: 0.43–0.98, *P* = 0.041) in individuals who had participated in ACS training programs, after adjusting for gender, age, education, job category, and monthly income. Furthermore, prior witness of ACS events in families (OR = 0.55, 95%CI: 0.38–0.79, *P* = 0.001) or personal experience of ACS (OR = 0.71, 95%CI: 0.49–1.00, *P* = 0.050) were also associated with decreased decision delay.

#### Mediation via symptom knowledge between past ACS exposure and decision delay

**Fig. 1:**
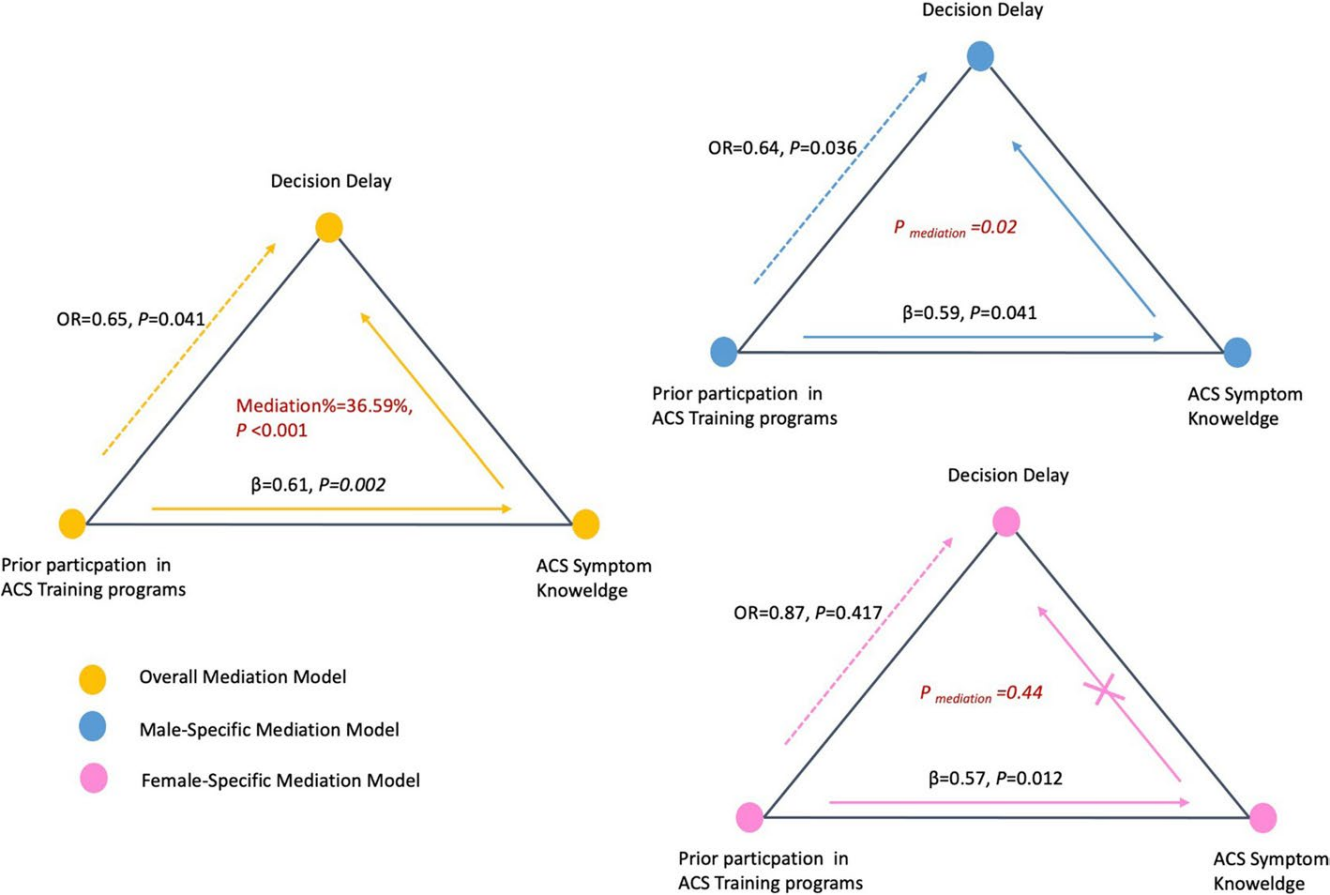
Mediation analysis: ACS symptom knowledge mediated the association between ACS training programs and decision delay

Mediation analysis (depicted in Fig. 1) demonstrated that the relationship between prior exposure to ACS training programs and decision delay was fully mediated by ACS symptom knowledge (mediation effect/total effect: 36.59%, *P* < 0.001), after controlling for gender, age, education, occupation and monthly income (as presented in Table 2). The proportions of the mediation effect were consistent when stratified by typical and atypical knowledge (*P* > 0.05).

**Table 2:** The mediating effect of symptom knowledge that link prior exposure to ACS to decision delay

Exposure	Model 1^a^	Model 2^b^	Model 3^c^	
	Total effect (OR,95%CI, *P*)	Assoc. Mediator (β,95%CI, *P*)	Mediation effect (%, *P*)	Direct effect (OR,95%CI, *P*)
Past ACS exposure	0.62 (0.44–0.88, *P* < 0.001)	0.49 (0.21–0.76, *P* < 0.001)	23.74 *P* < 0.0001	0.50 (0.43–0.85, *P* = 0.004)
(1) Prior exposure to training programs	0.65 (0.43–0.98, *P* = 0.041)	0.61 (0.23–0.98, *P =* 0.002)	36.59 *P* < 0.0001	0.40 (0.26–1.09, *P* = 0.110)
(2) Ever witness ACS in families	0.55 (0.38–0.79, *P* = 0.001)	0.50 (0.19–0.81, *P =* 0.001)	20.74 *P* < 0.0001	0.41 (0.20–0.81, *P* = 0.002)
(3) Ever personally experienced ACS	0.71 (0.49–1.00, *P* = 0.05)	0.31 (0.11–0.72, *P =* 0.001)	12.08 *P =* 0.72	0.58 (0.37–1.58, *P* = 0.621)

^a^ Model 1 assessed the total effect of past ACS exposure (as a whole) and its three elements on decision delay, with adjustments for gender, age, education, job category, and monthly income

^b^ Model 2 assessed the association between past ACS exposure (as a whole), its three elements and ACS symptom knowledge, with adjustments for gender, age, education, job category, and monthly income

^c^ Model 3 assessed the mediation via knowledge from past ACS exposure (as a whole), its three elements to decision delay, with adjustments for gender, age, education, job category, and monthly income. The estimate of mediation effect and its statistical significance was based on 1000 times of bootstrapping

#### Model 2: impact of ACS training programs and symptom knowledge

The results indicated that prior participation in ACS training programs was positively associated with higher ACS symptom knowledge (β = 0.49, 95%CI: 0.21–0.76, *P* < 0.001). Additionally, prior exposure to ACS events, either through personal experience (β = 0.31, 95%CI: 0.11–0.72, *P* = 0.001) or witnessing in families (β = 0.50, 95%CI: 0.19–0.81, P = 0.001), was also found to be positively associated with increased knowledge of ACS symptoms.

#### Mediating effect of knowledge linking ACS training to decision delay

The association between prior participation in ACS training programs and reduced decision delay was completely mediated by the increase in symptom knowledge (mediation effect = 36.59%, *P* < 0.001), after controlling for variables mentioned beforehand (Table 2). It’s also observed that the relationship between having witnessed ACS in families and decreased decision delay was partially mediated by knowledge, while the association between personal experience of ACS was not found to be mediated through ACS symptom knowledge.

### Mediation effect stratified by gender

The results of the stratified analysis by gender revealed heterogeneity in the mediation effect between prior participation in ACS training programs and decision delay in the male and female groups (P_for interaction_ < 0.01, see Table 3). The mediation effect was found to be statistically significant in the male group, with increased symptom knowledge fully mediating the effect of prior participation in training programs on decreased decision delay (*P* < 0.001). However, this was not observed in the female group (*P* = 0.44). This lack of mediation in the female group was due to the absence of a significant relationship between knowledge and decision delay. Although women gained a higher level of symptom knowledge (β = 0.57, *P* = 0.012) from prior participation in ACS training programs, this increased knowledge was not associated with a reduction in decision delay (*P* > 0.05). Furthermore, prior training was not found to be associated with decreased decision delay in the female group (OR = 0.87, 95%CI: 0.64–1.21, *P* = 0.417).

**Table 3:** Mediation effect on decision delay stratified by gender

Exposure	Gender	Model 1^a^	Model 2^b^	Model 3^c^	
		Total effect (OR,95%CI, *P*)	Assoc. Mediator (β,95%CI, *P*)	Mediation effect (%, *P*)	Direct effect (OR,95%CI, *P*)
Past ACS exposure	Male	0.54 (0.39–0.75, *P* < 0.001)	0.63 (0.15–1.11, *P =* 0.010)	17.39 *P* < 0.001	0.48 (0.33–0.70, *P* = 0.002)
	Female	0.69 (0.48–0.99, *P = 0.048*)	0.42 (0.08–0.75, *P* = 0.015)	24.01 *P* = 0.48	0.94 (0.73–1.22, *P* = 0.644)
(1) Prior exposure to training programs	Male	0.64 (0.42–0.98, *P* = 0.036)	0.59 (0.13–1.26, *P =* 0.041)	23.63 *P* = 0.02	0.70 (0.45–1.08, *P* = 0.103)
	Female	0.87 (0.64–1.21, *P* = 0.417)	0.57 (0.13–1.02, *P =* 0.012)	37.08 *P* = 0.44	0.93 (0.67–1.30, *P* = 0.667)
(2) Ever witness ACS in families	Male	0.64 (0.45–0.90, *P* = 0.011)	0.58 (0.04–1.12, *P =* 0.035)	18.38 *P* = 0.04	0.58 (0.42–0.80, *P <* 0.001)
	Female	0.71 (0.52–0.97, *P* = 0.0313)	0.43 (0.05–0.80, *P =* 0.026)	16.75 *P* = 0.08	0.80 (0.60–1.06, *P* = 0.111)
(3) Ever personally experienced ACS	Male	0.84 (0.56–1.27, *P* = 0.401)	0.56 (−0.07–1.19, *P =* 0.083)	29.99 *P =* 0.32	0.91 (0.60–1.40, *P* = 0.674)
	Female	1.11 (0.73–1.72, *P* = 0.633)	0.09 (−0.47–0.65, *P =* 0.757)	4.4 *P =* 0.84	1.13 (0.74–1.75, *P* = 0.587)

^a^ Model 1 assessed the total effect of past ACS exposure (as a whole) and its three elements on decision delay, stratified by gender, with adjustments for age, education, job category, and monthly income

^b^ Model 2 assessed the association between past ACS exposure (as a whole), its three elements and ACS symptom knowledge, stratified by gender, with adjustments for age, education, job category, and monthly income

^c^ Model 3 assessed the mediation via knowledge from past ACS exposure (as a whole), its three elements to decision delay, stratified by gender, with adjustments for age, education, job category, and monthly income. The estimate of mediation effect and its statistical significance was based on 1000 times of bootstrapping

## Discussion

The current study aimed to explore the relationship between prior exposure to ACS training programs and decision delay, with a focus on the mediating role of symptom knowledge. Despite previous research, there remains a limited understanding of the impact of past exposure to ACS on decision delay, particularly through the lens of symptom knowledge. Our community-based retrospective cohort study found that prior participation in ACS training programs was associated with decreased decision delay, partially mediated by increased symptom knowledge. However, our findings showed that the mediation effect was heterogeneous by gender, with a significant impact observed in the male group but not in the female group. These results suggest that while training can improve women’s knowledge of ACS prodromal symptoms, it may not necessarily reduce decision delay in this group. Further research is needed to address gender-specific barriers and to determine additional interventions beyond transferring knowledge alone.

The association between knowledge of prodromal symptoms of ACS and reduced decision delay has been well established in previous studies [10, 11, 23]. Based on this understanding, several intervention studies have been undertaken to reduce decision delay by improving knowledge of these symptoms [14, 15, 24–26]. However, despite an improvement in symptom knowledge through training, the efficacy of these interventions in reducing decision delay has been inconsistent [25, 27, 28], leading to uncertainties about the mediating role of symptom knowledge in the association between past ACS exposure and decision delay. The current study adds to the existing literature by demonstrating that participation in training programs, is associated with reduced decision delay via increased knowledge of prodromal symptoms. These results align with previous findings indicating that higher levels of knowledge may lead individuals to interpret symptoms as life-threatening warning signs [29]. Given the limited research in this area, our results support the potential benefit of training programs in reducing decision delay by raising awareness of prodromal symptoms of ACS.

It is also noteworthy to mention the gender differences in decision delay [17, 18]. While females have been found to have a longer delay before seeking help compared to males, they are also known to be more reluctant to seek emergency medical assistance [21]. However, the reasons for these differences have not been fully explored in previous studies [30–33]. Our study found that the anticipated mediation effect of symptom knowledge on decision delay was not evident in the female group. Despite higher levels of symptom knowledge among females, this did not correlate with a reduction in decision delay. This outcome suggests that while increasing knowledge might help reduce decision delays for males, alternative strategies may be necessary for females, who may face other gender-specific barriers. For instance, Zhang et al. identified Type D personality—a tendency towards negative affectivity and social inhibition—as a possible risk factor for delayed medical help-seeking in ACS among females [34]. Arrebola-Moreno et al. corroborated this, finding that Type D personality was linked to decision delays exclusively in females [35]. The prevalence of Type D personality in women could mean that it acts as an impediment to seeking timely emergency care, regardless of their symptom knowledge level. Previous interventions addressing emotional barriers in females corroborate this theory [36, 37]. Furthermore, the efficacy of ACS training programs for females may be augmented by tailoring to their high knowledge base, with a focus on advanced concepts, case studies, and practical applications. The significance of adapting ACS training to individuals with greater knowledge is underscored by recent research [38]. Addressing gender-specific barriers to prompt healthcare decision-making in females, particularly when they possess higher symptom knowledge yet hesitate to seek emergency medical services, necessitates a multifaceted approach. Solutions should include gender-sensitive education and awareness campaigns, challenging cultural norms, offering tailored health information, improving healthcare access, training healthcare providers to be sensitive to women’s unique health concerns, engaging in community outreach, involving family and social support networks, conducting research to understand specific barriers.

Our study has several strengths, including relatively a large sample size of community-based population in the capital city of China. To the best of our knowledge, this is the first community-based study in China that examined the interplay among training programs, symptom knowledge, decision delay and gender. Nevertheless, our study has limitations that require discussion. First, we assessed intentional decision delay as an alternative endpoint to real decision delay, which could introduce misclassification bias. However, the difference between intentional decision delay and real decision delay is likely non-differential and may bias true associations toward the null. Nevertheless, future studies involving ACS patients are expected to replicate our findings. Second, due to the cross-sectional design of our study where temporality cannot be guaranteed, it’s essential to note that the observed mediation may not necessarily indicate causation and should be interpreted cautiously. Therefore, further investigations with different study populations and designs are warranted.

## Conclusion

In conclusion, the present community-based retrospective cohort study conducted in Beijing, China, involved 22 communities and 1099 participants and found that prior participation in ACS training program was related to decision delay through the mediation of knowledge of ACS prodromal symptoms. The mediation process, however, was modified by gender, as it was only effective in the male group. These findings suggest that the promotion of ACS training programs and increased public awareness of ACS symptoms could be effective in reducing decision delay, particularly in males, while the effectiveness of such interventions for women may depend on addressing factors beyond symptom knowledge. Further research is needed to understand the reasons for the differences in the mediation effect by gender and to develop gender-specific strategies to improve the recognition and management of ACS.

### Supplementary Information

**Additional file 1.**

## Data Availability

The datasets generated and analyzed during the current study are not publicly available due to privacy, but are available from the corresponding author on reasonable request.
