# Supplementary material for: Gender disparities in the mediating role of symptom knowledge level in reducing acute coronary syndrome (ACS) decision delay: Findings from a community-based study in China
Source: BMC Emerg Med. 2023 Dec 16;23:146. doi: 10.1186/s12873-023-00916-5 (PMC10725594; doi:10.1186/s12873-023-00916-5)
Supplement: Supplementary file 1 — Additional file 1. [file 12873_2023_916_MOESM1_ESM.docx]

Supplementary Context

[The questionnaire used to investigate the survey’s Perception of and Response to Acute Coronary Syndrome (English Version) 1](#_Toc129613139)

[Supplementary Table 1: A multivariable logistic regression on characters associated with ACS prodromal symptoms knowledge level 5](#_Toc129613140)

**Supplementary Files_1**

# The questionnaire used to investigate the survey’s Perception of and Response to Acute Coronary Syndrome (English Version)

**1. General Information**

| 1.1 Name： 1.2 Gender：□Male □Female 1.3 Ethnicity： | | |
| --- | --- | --- |
| 1.4 Date of Birth | YY MM Date | 1.5 Phone： |
| 1.6 Education | □Before primary school □Primary School □Junior School □High School □Graduate □Postgraduate | |
| 1.7 Occupation | □Retiree □Household duties □Laid-off □Agriculture, forestry, animal husbandry and fishery workers □Private business owner □Administrative and managerial personnel □Workers □Sales and service staff □Professional and technical staff □Else (please specify) | |
| 1.8 Living Status | □Living in this area ≥5 year □ Living in this area <5 year | |
| 1.9 Medical Insurance Covered | □social medical insurance  □commercial health insurance  □Self-paying with no medical insurance  □Unsure  □Else (please specify) | |
| 1.10 Monthly income per family member (RMB) | - <1,000 □ 1,000-1,999 □ 2,000-2,999 - 3,000-4,999 □ 5,000-9,999 □ ≥10,000 | |

**2. Perception of and Response to Acute Coronary Syndrome**

| 2.1 Which might be the prodromal symptoms for heart attacks? | |
| --- | --- |
|  | Yes No Unsure |
| 2.1.1 Retrosternal compression, constrictive pain | - □ □ |
| 2.1.2 Retrosternal burning or heaviness | - □ □ |
| 2.1.3 Chest pain radiates to the neck, lower jaw, left arm or both upper arms | - □ □ |
| 2.1.4 Toothache | - □ □ |
| 2.1.5 The pain lasted longer than 10 minutes | - □ □ |
| 2.1.6 Chest discomfort with nausea or vomiting | - □ □ |
| 2.1.7 Chest discomfort with persistent shortness of breath or dyspnea | - □ □ |
| 2.2 What measures would you like to take in case you had an acute chest pain (multiple choices if you would take multiple measures)  □Stop ongoing activities and rest until symptoms disappear □Take nitroglycerin, quick effect jiuxin pills and other drugs  □Call emergency system if chest pain lasts longer than 5 minutes or if symptoms do not relieve with a nitroglycerin tablet  □Direct emergency department visit □Do not know what measures to take □Do nothing  □Else (please specify) | |
| 2.3 What should be informed when calling emergency system (multiple choices if you would inform multiple pieces of info.)  □Name, age and gender of the sufferers □Detailed address to locate the sufferers □Symptoms of this attack □History of symptoms alike □Rescue measures that have been taken | |
| 2.4 What might prevent you from instant utilization of emergency medical services when you experienced an acute chest pain lasting over 5minutes (multiple choices if needed)  □Assume the symptom tolerable □ Inconvenience for medical services □Concern about medical costs □Do not know how to seek help □ I will not let things mentioned above happen. Instead, I will take seek for instant medical help | |
| 2.5 Will you seek for instant medical help in case your families/friends experienced an acute chest pain?  □ Yes □ No □Unsure  □ It depends. Unless something happened, I would help (please specify the circumstances) | |
| 2.6 Do you agree that instant medical services would save the lives of those who experienced acute chest pain?  □ Yes □ No | |

**3. Needs for a health education campaign**

| 3.1 Have you ever been exposed to a training program / advertisements /propaganda about acute coronary syndrome>  □ Yes □ No □ Unsure |
| --- |
| 3.2 What would you like to know about acute coronary syndrome (multiple choices if needed)  □Prodromal symptoms of acute chest pain □Ideal response measure to acute chest pain □Preventive measures to acute chest pain □Treatment to acute chest pain □ Else (please specify) |
| 3.3 What channels would you like to be promoted (multiple choices if needed)  □Talk □Television □Pamphlets/Posters □Radio/Podcast □Newspaper/Magazine □Internet via cellphone/PC □Else (please specify) |

**4. Personal history**

| 4.1 Have you ever been exposed to acute chest pain?  □ Yes □ No □ Unsure | |
| --- | --- |
| 4.2 Do you have the following diseases? | |
|  | Yes No Unsure |
| 4.2.1 acute myocardial infarction | - □ □ |
| 4.2.2 unstable angina pectoris | - □ □ |
| 4.2.3 Hypertension | - □ □ |
| 4.2.4 Hyperlipidemia | - □ □ |
| 4.2.5 Diabetes | - □ □ |
| 4.3 Do you have any family who had ever been exposed to chest pain?  □ Yes (specify the relationship)_____ □ No □ Unsure | |
| 4.4 Do you smoke?  □ currently □ once and now quit □Never | |
| 4.5 Do you drink?  □ currently □ once and now quit □Never | |
| 4.6 Do you take Moderate or high intensity physical activity and at a frequency higher than 5 days/week and 30mintues /day  □ Yes □ No □ Unclear | |

**Supplementary Table 1**

# Supplementary Table 1: A multivariable logistic regression on characters associated with ACS prodromal symptoms knowledge level

|  | Overall |  |  | Male |  |  | Female |  |
| --- | --- | --- | --- | --- | --- | --- | --- | --- |
|  | β, OR, 95%CI | P |  | β, OR, 95%CI | P |  | β, OR, 95%CI | P |
| Age |  |  |  |  |  |  |  |  |
| 18-25 | 0.36,1.43(0.83,2.46) | 0.195 |  | -0.12,0.89(0.36,2.15) | 0.798 |  | 0.75,2.11(0.99,4.54) | 0.055 |
| 25-35 | 0.48,1.62(1.03,2.53) | *0.035 |  | 0.47,1.60(0.84,3.08) | 0.153 |  | 0.50,1.65(0.83,3.24) | 0.150 |
| 35-45 | 0.01,1.01(0.63,1.61) | 0.972 |  | -0.19,0.82(0.44,1.54) | 0.541 |  | 0.31,1.36(0.63,2.96) | 0.436 |
| >=45 | 1 (ref) |  |  | 1(ref) |  |  | 1(ref) |  |
| Occupation |  |  |  |  |  |  |  |  |
| Retired | 0.01,1.01(0.49,2.09) | 0.989 |  | -0.77,0.46(0.12,1.69) | 0.244 |  | 0.49,1.63(0.60,4.49) | 0.344 |
| Self-employed/Unemployed | -0.86,0.42(0.19,0.91) | *0.029 |  | -1.75,0.17(0.04,0.62) | *0.010 |  | -0.26,0.77(0.28,2.15) | 0.619 |
| Manager | -0.17,0.85(0.52,1.38) | 0.499 |  | -1.02,0.36(0.17,0.75) | **0.007 |  | 0.54,1.72(0.87,3.46) | 0.125 |
| Worker | 0.33,1.39(0.82,2.36) | 0.220 |  | -0.65,0.52(0.24,1.11) | 0.092 |  | 1.38,3.97(1.68,10.00) | **0.002 |
| Salesman/Service | -0.06,0.94(0.45,2.01) | 0.872 |  | -0.19,0.83(0.28,2.54) | 0.741 |  | 0.10,1.10(0.38,3.37) | 0.859 |
| Technical | 1 (ref) |  |  | 1(ref) |  |  | 1(ref) |  |
| Other | 0.18,1.19(0.70,2.04) | 0.515 |  | -0.42,0.66(0.26,1.64) | 0.369 |  | 0.66,1.93(0.98,3.84) | 0.059 |
| Monthly income per capita |  |  |  |  |  |  |  |  |
| <=3000 | 1 (ref) |  |  | 1(ref) |  |  | 1(ref) |  |
| 3000-5000 | 0.32,1.37(0.92,2.05) | 0.118 |  | -0.36,0.70(0.37,1.30) | 0.257 |  | 0.80,2.22(1.26,3.96) | **0.006 |
| 5000-10000 | 0.41,1.51(0.99,2.32) | 0.057 |  | -0.15,0.86((0.44,1.67) | 0.663 |  | 0.82,2.26(1.23,4.23) | **0.010 |
| >=10000 | 0.56,1.75(1.03,3.00) | *0.039 |  | -0.14,0.87(0.27,3.04) | 0.739 |  | 1.04,2.83(1.37,6.02) | **0.006 |
| Medical insurance |  |  |  |  |  |  |  |  |
| Yes | 1 (ref) |  |  | 1(ref) |  |  | 1(ref) |  |
| No | -1.22,0.29(0.13,0.64) | *0.002 |  | -1.95,0.14(0.01,0.99) | 0.089 |  | -1.10,0.33(0.13,0.86) | *0.023 |
|  |  |  |  |  |  |  |  |  |
| Witness to ACS in families |  |  |  |  |  |  |  |  |
| Yes | 0.44,1.56(1.10,2.24) | **0.015 |  | 0.25,1.29(0.76,2.20) | 0.352 |  | 0.50,1.58(0.95,2.69) | 0.082 |
| No | 1 (ref) |  |  | 1(ref) |  |  | 1(ref) |  |
| Unclear | -0.35,0.71(0.46,1.09) | 0.118 |  | -0.13,0.88(0.44,1.75) | 0.717 |  | -0.42,0.65(0.36,1.20) | 0.166 |
| Prior Participation in ACS training programs |  |  |  |  |  |  |  |  |
| Yes | 0.65,1.91(1.24,3.01) | **0.004 |  | 0.26,1.30(0.68,2.54) | 0.436 |  | 1.06,2.87(1.51,5.83) | **0.002 |
| No | 1 (ref) |  |  | 1(ref) |  |  | 1(ref) |  |
| Unclear | -0.85,0.43(0.23,0.77) | **0.005 |  | -1.55,0.21(0.06,0.58) | **0.005 |  | -0.54,0.58(0.26,1.33) | 0.193 |
| Knowledge on instant EMS utilization benefits |  |  |  |  |  |  |  |  |
| Yes | 0.82,2.27(1.50,3.45) | ***<0.001 |  | 1.15,3.17(1.76,5.86) | ***<0.001 |  | 0.62,1.86(0.96,3.56) | 0.062 |
| No | 1 (ref) |  |  | 1(ref) |  |  | 1(ref) |  |

*** P<0.001, ** P<0.01, *P<0.05;

OR reported based on models adjusted for social economic status (gender, age, education, income, medical insurance), experience (family history, previous training, self-experienced chest pain), attitude, clinical status and behavior (diabetes, dyslipidemia, hypertension and smoking) and for variables not statistically significant in any groups were not listed.
